# Diversity of the genus *Sugiyamaella* and description of two new species from rotting wood in China

**DOI:** 10.3897/mycokeys.77.60077

**Published:** 2021-01-12

**Authors:** Cheng-Feng Shi, Kai-Hong Zhang, Chun-Yue Chai, Zhen-Li Yan, Feng-Li Hui

**Affiliations:** 1 School of Life Science and Technology, Nanyang Normal University, Nanyang 473061, China Nanyang Normal University Nanyang China; 2 State Key Laboratory of Motor Vehicle Biofuel Technology, Henan Tianguan Enterprise Group Co., Ltd., Nanyang 473000, China Henan Tianguan Enterprise Group Co., Ltd. Nanyang China

**Keywords:** Phylogeny, rotted wood-inhabiting yeast, *
Sugiyamaella
*, taxonomy, Trichomonascaceae

## Abstract

Species of the genus *Sugiyamaella* (Trichomonascaceae, Saccharomycetales), found in rotting wood in China, were investigated using morphology and the molecular phylogeny of a combined ITS and nrLSU dataset. Nine taxa were collected in China: two were new species (viz. *Sugiyamaella chuxiong***sp. nov.** and *S. yunanensis***sp. nov.**) and seven were known species, *S. americana*, *S. ayubii*, *S. novakii*, *S. paludigena*, *S. valenteae*, *S. valdiviana* and *S. xiaguanensis*. The two new species are illustrated and their morphology and phylogenetic relationships with other *Sugiyamaella* species are discussed. Our results indicate a potentially great diversity of *Sugiyamaella* spp. inhabiting rotting wood in China just waiting to be discovered.

## Introduction

*Sugiyamaella* Kurtzman & Robnett (2007) is typified by *Sugiyamaella smithiae*, which was initially classified in the genus *Stephanoascus* (Giménez-Jurado et al. 1994). The genus *Sugiyamaella* belongs to the family Trichomonascaceae in the order Saccharomycetales and is closely related to the genera *Trichomonascus*, *Wickerhamiella* and *Zygoascus*, based on multigene phylogenetic analyses of LSU, MtSm and *COXII* nucleotide sequences (Kurtzman and Robnett 2007; Péter et al. 2012) .

Kurtzman (2011) accepted four species in *Sugiyamaella* and proposed a key for this genus, based mainly on the reactions on standard growth and fermentation tests. Subsequently, *S. ayubii*, *S. bahiana*, *S. bonitensis*, *S. carassensis*, *S. ligni*, *S. mastotermitis*, *S. trypani*, *S. valenteae*, *S. xiaguanensis*, *S. xylolytica* and *S. xylanicola* were added to this genus (Morais et al. 2013; Handel et al. 2016; Sena et al. 2017; Huang et al. 2018; Crous et al. 2019). Within the same time frame, 14 *Candida* species in this clade were transferred to the genus *Sugiyamaella* as new combinations, based on their phylogeny (Urbina et al. 2013; Handel et al. 2016). Thus, 29 species were included in this genus before our study, 25 were asexual morphs and four had known ascosporic states, viz. *S. americana*, *S. chiloensis*, *S. japonica* and *S. smithiae* (Kurtzman 2007; Kurtzman and Robnett 2007; Morais et al. 2013; Handel et al. 2016; Sena et al. 2017; Huang et al. 2018; Crous et al. 2019). Morphologically, the sexual morph of *Sugiyamaella* is characterised by the production of globose to ellipsoidal asci with a single ellipsoidal or bacilliform ascospore. The asexual morph is characterised by multilateral budding and formation of blastoconidia. The other useful morphological feature is that pseudohyphae and true hyphae are commonly formed (Kurtzman and Robnett 2007; Kurtzman 2011; Sena et al. 2017).

The members of *Sugiyamaella* have been described in association with insects. They were isolated either directly from wood-ingesting insects and insect frass or from common insect habitats, such as rotting wood, forest soil, mushrooms and peat (Kurtzman 2007; Wang et al. 2010; Kurtzman 2011; Morais et al. 2013; Handel et al. 2016; Sena et al. 2017; Huang et al. 2018). Significantly, most species of *Sugiyamaella* have been reported as potential xylanase producers (Morais et al. 2013; Lara et al. 2014; Handel et al. 2016; Sena et al. 2017). Several species of *Sugiyamaella*, including *S. bahiana*, *S. bonitensis*, *S. boreocaroliniensis*, *S. lignohabitans*, *S. valenteae*, *S. xylanicola* and *S. xylolytica*, possess the ability to ferment d-xylose, which gives them economic potential for production of bioethanol and/or xylitol from plant waste residues (Morais et al. 2013; Sena et al. 2017). Therefore, *Sugiyamaella* species are important, not only for their wood-decaying activity, but also for their potential application in food, medicine and biofuels.

*Sugiyamaella* has a worldwide distribution and most of its species were originally found in Europe, North America and South America (Kurtzman 2007; Kurtzman 2011; Morais et al. 2013; Sena et al. 2017). The genus has not received as much attention in Asia, except for two novel species described from Japan (Kurtzman 2007; Kurtzman 2011). In China, two novel taxa have been described (Wang et al. 2010; Huang et al. 2018). To date, only four *Sugiyamaella* species have been reported in China, namely *S. lignohabitans*, *S. qingdaonensis*, *S. smithiae* and *S. xiaguanensis* (Wang et al. 2010; Zhai et al. 2019; Huang et al. 2018). In this study, we collected rotting wood samples from Yunnan Province in China. After isolation and examination, two new species and seven known species of *Sugiyamaella* were identified, based on morphology and molecular phylogenetic analysis, increasing the species diversity of *Sugiyamaella* in China.

## Materials and methods

### Sample collection, morphological studies and isolation

Rotting wood samples were collected in two areas of Yunnan Province, China. The areas were located in the Xishuangbanna Primeval Forest Park of Jinghong (21°98'N, 100°88'E) and Zixi Mountain of Chuxiong (25°03'N, 101°41'E). The predominant vegetation is characterised as tropical and subtropical forest biome. The climate is hot and humid, with annual precipitation between 1,000 to 1,600 mm and an average temperature that ranges from 14.8 to 21.9 °C. Sixty decayed wood samples were collected during July to August in 2016–2018. The samples were stored in sterile plastic bags and transported under refrigeration to the laboratory over a period of no more than 24 h. The yeast strains were isolated from rotting wood samples in accordance with the methods described by Morais et al. (2013) and Lopes et al. (2016). Each sample (1 g) was added to 20 ml sterile d-xylose medium (yeast nitrogen base 0.67%, d-xylose 0.5% and chloramphenicol 0.02%, pH 5.0 ± 0.2) in a 150 ml Erlenmeyer flask and then cultured for 3–10 days on a rotary shaker. Subsequently, 0.1 ml aliquots of the enrichment culture and appropriate decimal dilutions were spread on d-xylose agar plates and then incubated at 25 °C for 3–4 days. Different yeast colony morphotypes were then isolated by repeated plating on yeast extract-malt extract (YM) agar (1% glucose, 0.5% peptone, 0.3% yeast extract and 0.3% malt extract, pH 5.0 ± 0.2) and then stored on YM agar slants at 4 °C or in 15% glycerol at -80 °C.

The morphological, physiological and biochemical properties were determined according to those used by Kurtzman et al. (2011). The beginning of the sexual stage was determined by incubating single or mixed cultures of each of the two strains on corn-meal (CM) agar, 5% malt extract (ME) agar, dilute (1:9) V8 agar or yeast carbon base plus 0.01% ammonium sulphate (YCBAS) agar at 15 and 25 °C for 6 weeks (Kurtzman 2007; Huang et al. 2018). The assimilation of carbon and nitrogen compounds and related growth requirements were tested at 25 °C. The effects of temperature from 25–40 °C were examined in liquid and agar plate cultures.

### DNA extraction, PCR amplification and sequencing

Genomic DNA was extracted from the yeast using an Ezup Column Yeast Genomic DNA Purification Kit, according to the manufacturer’s instructions (Sangon Biotech, Shanghai, China). The nuc rDNA ITS1-5.8S-ITS2 (ITS) region was amplified using primer pairs ITS1/ITS4 (White et al. 1990). The D1/D2 domain of nrLSU rDNA (nrLSU) was amplified using the primer pairs NL1/NL4 (Kurtzman and Robnett 1998). The following thermal profile was used to amplify the ITS and D1/D2 nrLSU regions: an initial denaturation step of 2 min at 95 °C; followed by 35 cycles of 30 s at 95 °C, 30 s at 51 °C and 40 s at 72 °C; with a final extension of 10 min at 72 °C (Liu et al. 2016). PCR products were directly purified and sequenced by Sangon Biotech Inc. (Shanghai, China). We confirmed the identity and accuracy of the resulting sequences by assembling them using BioEdit and comparing them to sequences in GenBank (Hall 1999). The sequences were then submitted to GenBank (https://www.ncbi.nlm.nih.gov/genbank/; Table 1).

### Phylogenetic analysis

The sequences obtained from this study and the reference sequences downloaded from GenBank (Table 1) were aligned using MAFFT v. 6 (Katoh and Toh 2010) and manually edited using MEGA7 (Kumar et al. 2016). The best-fit nucleotide substitution models for each gene were selected using jModelTest v2.1.7 (Darriba et al. 2012) according to the Akaike Information Criterion. Phylogenetic analyses of combined gene regions (ITS and nrLSU) were performed using MEGA7 for Maximum Parsimony (MP) analysis (Kumar et al. 2016) and PhyML v3.0 for Maximum Likelihood (ML) analysis (Guindon et al. 2010). *Schizosaccharomyces pombe*NRRL Y-12796 was chosen as the outgroup after consulting Morais et al. (2013) and Sena et al. (2017).

**Table 1.** Sequences used in molecular phylogenetic analysis. Entries in bold are newly generated for this study.

Species	Strain	Locality	Sample	ITS	D1/D2
*Sugiyamaella americana*	NRRL YB-2067^T^	USA	Frass	NR_137759	DQ438193
***S. americana***	**NYNU 17714**	**China**	**Rotting wood**	**MT965698**	**MT965699**
*S. ayubii*	CBS 14108^T^	Brazil	Rotting wood	NR_155796	KR184132
***S. ayubii***	**NYNU 177171**	**China**	**Rotting wood**	**MT965704**	**MT965705**
*S. bahiana*	CBS 13474^T^	Brazil	Rotting wood	NR_155810	KC959941
*S. bonitensis*	CBS 14270^T^	Brazil	Rotting wood	NR_155798	KT006004
*S. boreocaroliniensis*	NRRL YB-1835^T^	USA	Frass	NR_165963	DQ438221
*S. bullrunensis*	CBS 11840^T^	USA	Insect	NR_111543	HM208601
*S. castrensis*	NRRL Y-17329^T^	Chile	Rotting wood	NR_111229	DQ438195
*S. carassensis*	CBS 14107^T^	Brazil	Rotting wood	NR_155808	KX550111
*S. chiloensis*	NRRL Y-17643^T^	Chile	Rotted wood	DQ911454	DQ438217
*S. floridensis*	NRRL YB-3827^T^	USA	Frass	NR_111230	DQ438222
*S. grinbergsii*	NRRL Y-27117^T^	Chile	Insect	KY102116	DQ438199
*S. japonica*	NRRL YB-2798^T^	Japan	Frass	NR_111239	DQ438202
*S. ligni*	CBS 13482^T^	Brazil	Rotting wood	KX550112	KX550112
*S. lignohabitans*	NRRL YB-1473^T^	USA	Decayed log	NR_119622	DQ438198
*S. marionensis*	NRRL YB-1336^T^	USA	Decayed log	NR_111237	DQ438197
*S. marilandica*	NRRL YB-1847^T^	USA	Frass	NR_165965	DQ438219
*S. mastotermitis*	CBS 14182^T^	Berlin	Termite	NR_156606	KU883286
*S. neomexicana*	CBS 10349^T^	USA	Frass	NR_165966	DQ438201
*S. novakii*	NRRL Y-27346^T^	Hungary	Rotting wood	NR_111235	DQ438196
***S. novakii***	**NYNU 17778**	**China**	**Rotting wood**	**MT965702**	**MT965703**
*S. paludigena*	NRRL Y-12697^T^	Russia	Peat	NR_111236	DQ438194
***S. paludigena***	**NYNU 1771**	**China**	**Rotting wood**	**MT965696**	**MT965697**
***S. paludigena***	**NYNU 177116**	**China**	**Rotting wood**	**MT966075**	**MT966074**
*S. pinicola*	CBS 10348^T^	USA	Frass	NR_165967	DQ438200
*S. qingdaonensis*	CBS 11390^T^	China	Rotting wood	NR_151806	FJ613527
*S. smithiae*	NRRL Y-17850^T^	Brazil	Soil	DQ911455	DQ438218
*S. trypani*	CBS 15876^T^	Poland	Soil	MK388412	MK387312
*S. valdiviana*	NRRL Y-7791^T^	Chile	Rotting wood	NR_111544	DQ438220
***S. valdiviana***	**NYNU17755**	**China**	**Rotting wood**	**MT965700**	**MT965701**
*S. valenteae*	CBS 14109^T^	Brazil	Rotting wood	NR_155797	KT005999
***S. valenteae***	**NYNU 17795**	**China**	**Rotting wood**	**MT965706**	**MT965707**
*S. xiaguanensis*	NYNU 161041^T^	China	Rotting wood	KY213802	KY213817
***S. xiaguanensis***	**NYNU 17753**	**China**	**Rotting wood**	**MT969346**	**MT969344**
*S. xylanicola*	CBS 12683^T^	Brazil	Rotting wood	KC493642	KC493642
*S. xylolytica*	CBS 13493^T^	Brazil	Rotting wood	KU214874	KF889433
***S. yunanensis***	**NYNU 161059^T^**	**China**	**Rotting wood**	**MT257259**	**MT257257**
***S. yunanensis***	**NYNU 16113**	**China**	**Rotting wood**	**MT257256**	**MT257261**
*Candida* sp.	W370	Taiwan	Forest soil	JN581120	JN581115
*Candida* sp.	GA2M09	Taiwan	Mushroom	FJ873591	FJ873521
***S. chuxiongensis***	**NYNU 181038^T^**	China	Rotting wood	**MK682800**	**MK682795**
***S. chuxiongensis***	**NYNU 18521**	China	Rotting wood	**MT257260**	**MT257255**
***S. chuxiongensis***	**NYNU 18634**	China	Rotting wood	**MT257258**	**MT257262**
*Schizosaccharomyces pombe*	NRRL Y-12796^T^	–	–	KY105378	AY048171

Abbreviations: **CBS**: CBS-KNAW Collections, Westerdijk Fungal Biodiversity Institute, Utrecht, The Netherlands; **NRRL**: Agricultural Research Service Culture Collection, Peoria, IL, USA; **NYNU**: Microbiology Lab, Nanyang Normal University, Henan, China; **T**: type strain.

Maximum Parsimony analysis was performed using a heuristic search option with tree-bisection reconnection (TBR) branch swapping (Nei and Kumar 2000) and 1,000 random sequence additions. Maximum Likelihood analysis was performed using GTR+I+G models for each partition (Nei and Kumar 2000) and a proportion of invariant sites with 1000 rapid bootstrap replicates. The phylogenies from MP and ML analyses were displayed using Mega7 and FigTree v1.4.3 (Rambaut 2016), respectively. Bootstrap support values ≥ 50% are shown at the nodes.

## Results

### Phylogenetic analyses

**Figure 1.**
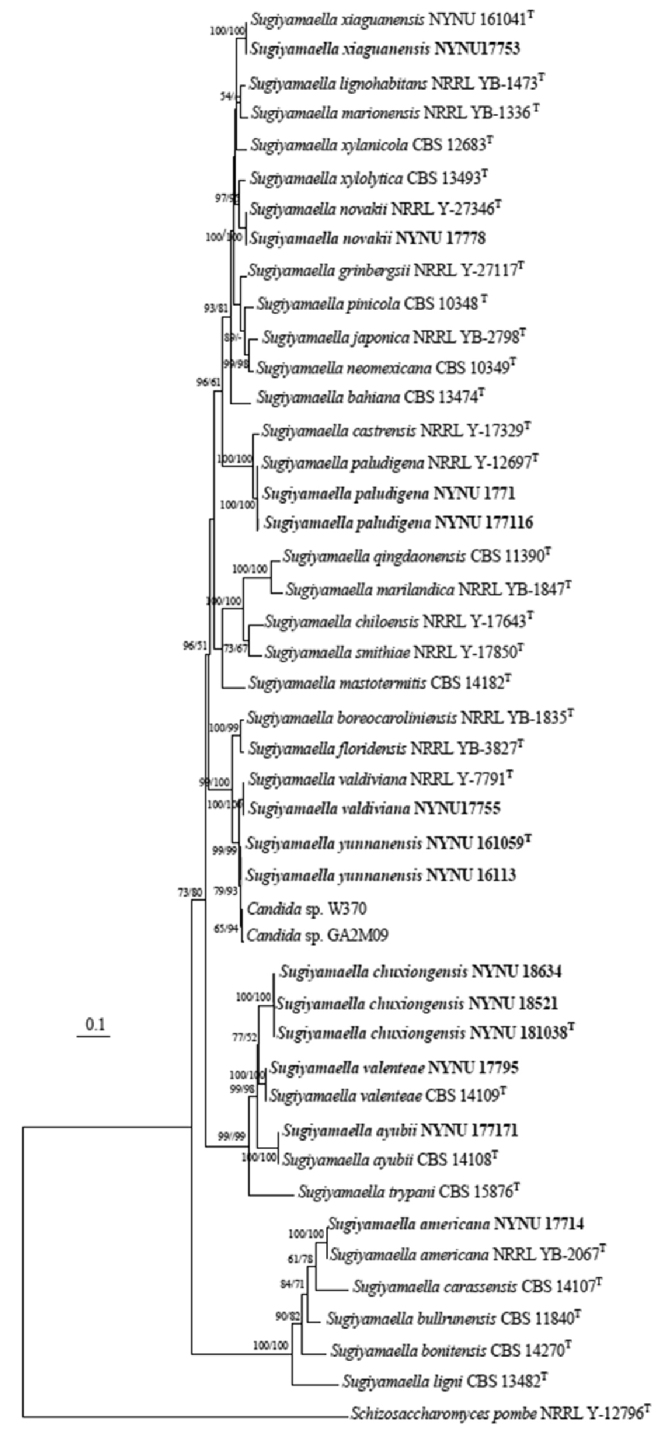
Maximum Likelihood phylogenetic tree of *Sugiyamaella* inferred from the combined ITS and nrLSU dataset and rooted with *Schizosaccharomyces pombe*NRRL Y-12796. The ML and MP bootstrap support values above 50% are shown at the first and second positions, respectively. Newly-sequenced collections are in black boldface.

The alignment was based on the combined nuclear dataset (ITS and nrLSU), included 31 taxa and one outgroup taxon (*Schizosaccharomyces pombe*NRRL Y-12796) and was comprised of 976 characters including gaps (385 for ITS and 591 for nrLSU) in the aligned matrix. Of these characters, 452 were constant, 164 variable characters were parsimony-uninformative and 360 characters were parsimony-informative. The heuristic search, using MP analysis, generated the most parsimonious tree (TL = 1627, CI = 0.457, RI = 0.766, RC = 0.394). The best model applied in the ML analysis was GTR+I+G. The ML analysis yielded a best scoring tree with a final optimisation likelihood value of –8651.84. Two methods for phylogenetic tree construction resulted in a similar topology. Therefore, only the best scoring PhyML tree is shown with BS and BT values simultaneously in Fig. 1.

From the phylogenetic tree (Fig. 1), seven known species, including *S. americana*, *S. ayubii*, *S. novakii*, *S. paludigena*, *S. valenteae*, *S. valdiviana* and *S. xiaguanensis*, were absorbed in the genus *Sugiyamaella*. *Sugiyamaella chuxiong* and *S. yunanensis* are new to science, based on the distinct and well-supported molecular phylogenetic placement and morphological differences with their closest described relatives. Phylogenetically, strains of *S. chuxiong* formed a unique lineage with 100% bootstrap support, while *S. yunanensis* was closely related to *S. valdiviana* with high bootstrap support (99%). The collection, labelled *Candida* sp. (W370) from Taiwan, clustered together with *S. yunanensis* and another species labelled *Candida* sp. (GA2M09) from mushroom.

### Taxonomy

#### Sugiyamaella yunanensis

Taxon classificationFungiSaccharomycetalesTrichomonascaceae

C.Y. Chai & F.L. Hui sp. nov.

32E2FFB9-533C-5D1B-BF8E-45A0B8EF49F8

835004

Figure 2

##### Type.

China, Yunnan Province, Jinghong City, Mengyang Town, in rotting wood from a tropical rainforest, July 2016, K.F. Liu & L. Zhang (holotype NYNU 161059^T^, culture ex-type CBS 14701).

##### Etymology.

The species name *yunanensis* (N.L. fem. adj.) refers to the geographical origin of the type strain of this species.

##### Description.

**Figure 2.**
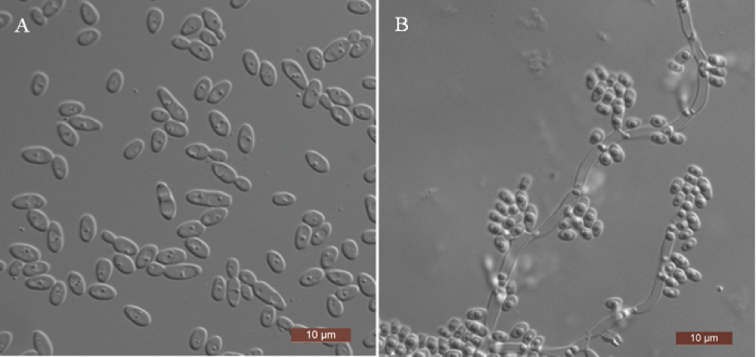
Morphology of *S. yunanensis***A** budding cells after 3 days in YM broth at 25 °C **B** hyphae and blastoconidia on corn-meal agar after 7 days at 25 °C. Scale bars: 10 μm.

The cells are ovoid to elongate (2.5–5.5 × 3–7.5 μm) and occur singly or in pairs after being placed in YM broth for 3 days at 25 °C (Fig. 2A). Budding is multilateral. After 3 days of growth on YM agar at 25 °C, the colonies are white to cream-coloured, buttery and smooth, with entire margins. After 7 days at 25 °C on a Dalmau plate culture with CM agar, hyphae and blastoconidia are formed (Fig. 2B). Asci or signs of conjugation were not observed on sporulation media. Glucose and d-xylose are weakly fermented. Glucose, galactose, l-sorbose, d-glucosamine, d-xylose, l-arabinose, d-arabinose, sucrose, maltose, trehalose, methyl α-d-glucoside, cellobiose, salicin, arbutin, melibiose, raffinose, inulin, ribitol, d-glucitol, d-mannitol, d-glucono-1, 5-lactone, 2-keto-d-gluconate, d-gluconate, d-glucuronate, dl-lactate, succinate, citrate and ethanol are assimilated. No growth was observed in d-ribose, l-rhamnose, lactose, melezitose, glycerol, erythritol, xylitol, galactitol, myo-inositol or methanol. In nitrogen-assimilation tests, growth is present on nitrate, nitrite, l-lysine and glucosamine, while growth is absent on ethylamine, cadaverine, creatine, creatinine, imidazole and d-tryptophan. Growth is observed at 37 °C, but not at 40 °C. Growth in the presence of 0.01% cycloheximide is present, but growth in the presence of 10% sodium chloride (NaCl) with 5% glucose and 1% acetic acid is absent. Starch-like compounds are not produced. Urease activity and diazonium blue B reactions are negative.

##### Additional isolate examined.

China, Yunnan Province, Jinghong City, Mengyang Town, in rotting wood from a tropical rainforest, July 2016, K.F. Liu & L. Zhang, NYNU 16113.

##### GenBank accession numbers.

holotype NYNU 161059^T^ (ITS: MT257259; nrLSU D1/D2: MT257257); additional isolate NYNU 16113 (ITS: MT257256; nrLSU D1/D2: MT257261).

##### Notes.

Two isolates, representing *S. yunanensis*, are retrieved in a well-supported clade and appear most closely related to *S. valdiviana* (Fig. 1). *Sugiyamaella yunanensis* can be distinguished from *S. valdiviana*, based on ITS and nrLSU D1/D2 loci (6/510 in ITS and 7/557 in nrLSU D1/D2). Physiologically, *S. yunanensis* differs from *S. valdiviana* by its ability to assimilate inulin and dl-lactate and its inability to assimilate melezitose, glycerol and myo-inositol. Additionally, *S. valdiviana* grows in the presence of 0.1% cycloheximide, while *S. yunanensis* does not (Kurtzman 2007).

#### Sugiyamaella chuxiongensis

Taxon classificationFungiSaccharomycetalesTrichomonascaceae

C.Y. Chai & F.L. Hui sp. nov.

5534E525-864B-5D30-B0A7-EA442993E812

835005

Figure 3

##### Type.

China, Yunnan Province, Chuxiong City, Zixi Town, in rotting wood from Zixi Mountain, August 2018, K.F. Liu & Z.W. Xi (holotype NYNU 181038^T^, culture ex-type CBS 16006, CICC 33361).

##### Description.

**Figure 3.**
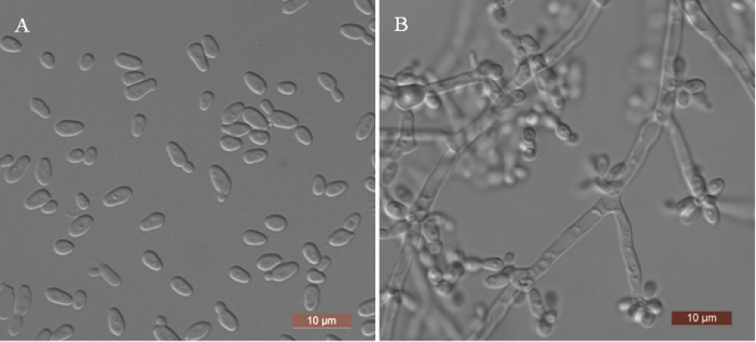
Morphology of *S. chuxiongensis***A** budding cells after 3 days in YM broth at 25 °C **B** hyphae and blastoconidia on corn-meal agar after 7 days at 25 °C. Scale bars: 10 μm.

The cells are ovoid to elongate (2.5–4 × 3–4.5 μm) and occur singly or in pairs after growth in a YM broth for 3 days at 25 °C (Fig. 3A). Budding is multilateral. After 3 days of growth on YM agar at 25 °C, the colonies are white to cream-coloured, buttery and smooth with entire margins. After 7 days at 25 °C, on a Dalmau plate culture with CM agar, hyphae and blastoconidia are formed (Fig. 3B). Asci or signs of conjugation were not observed on sporulation media. Fermentation of sugars is absent. Glucose, galactose, l-sorbose, d-glucosamine, d-ribose, d-xylose, l-arabinose, d-arabinose, sucrose, maltose, trehalose, methyl α-d-glucoside, cellobiose, salicin, arbutin, melibiose, raffinose, melezitose, inulin, erythritol, ribitol, xylitol, d-glucitol, d-mannitol, d-glucitol, d-mannitol, galactitol, myo-inositol, 2-keto-d-gluconate, succinate, citrate and ethanol are assimilated. No growth was observed in l-rhamnose, lactose, glycerol, d-gluconate, dl-lactate or methanol. In nitrogen-assimilation tests, growth is present on nitrate, nitrite, ethylamine, cadaverine, creatine, creatinine, glucosamine and d-tryptophan, while growth is absent on l-lysine and imidazole. Growth was observed at 35 °C, but not at 37 °C. Growth in the presence of 0.1% cycloheximide, 10% NaCl with 5% glucose and 1% acetic acid is present, but growth in the presence of 16% NaCl with 5% glucose is absent. Starch-like compounds are not produced. Urease activity and diazonium blue B reactions are negative.

##### Additional isolates examined.

China, Yunnan Province, Chuxiong City, Zixi Town, in rotting wood from Zixi Mountain, August 2018, K.F. Liu & Z.W. Xi, NYNU 18521, NYNU 18634.

##### GenBank accession numbers.

holotype NYNU 181038^T^ (ITS: MK682800; nrLSU D1/D2: MK682795); additional isolates NYNU 18521 (ITS: MT257260; nrLSU D1/D2: MT257255) and NYNU 18634 (ITS: MT257258; nrLSU D1/D2: MT257262).

##### Notes.

We generated sequences for three isolates of *S. chuxiong*, NYNU 18521, NYNU 181038 and NYNU 18634. This new species is phylogenetically most closely related to *S. valenteae* and *S. ayubii* (Fig. 1). *Sugiyamaella chuxiong* can be distinguished from *S. valenteae*, based on ITS and nrLSU D1/D2 loci (33/454 in ITS and 15/513 in nrLSU D1/D2) and from *S. ayubii*, based on ITS and nrLSU D1/D2 (42/499 in ITS and 35/565 in nrLSU D1/D2). Physiologically, *S. chuxiong* can be differentiated from *S. valenteae* by its ability to assimilate d-arabinose, sucrose, salicin, melibiose, raffinose, melezitose and inulin and its inability to ferment glucose and grow at 37 °C (Sena et al. 2017). Similarly, the ability to assimilate salicin, inulin, erythritol and galactitol and the inability to assimilate l-rhamnose are the primary differences between *S. chuxiong* and *S. ayubii*. Additionally, *S. ayubii* can ferment glucose, while *S. chuxiong* cannot (Sena et al. 2017).

## Discussion

In this study, nine *Sugiyamaella* species were identified, based on morphological and molecular phylogenetic analyses. All species were isolated from rotting wood collected in Yunnan Province, China. As a result, *S. chuxiong* and *S. yunanensis* are proposed as new species in *Sugiyamaella* for their distinct phylogenic positions and distinctive physiological traits. In addition, identification of seven known species of *Sugiyamaella*, *S. americana*, *S. ayubii*, *S. novakii*, *S. paludigena*, *S. valenteae*, *S. valdiviana* and *S. xiaguanensis* were clearly distinguished by both morphological and molecular approaches.

Molecular phylogeny studies on *Sugiyamaella* and related genera have been carried out recently (Handel et al. 2016; Sena et al. 2017). Handel et al. (2016) determined that *Sugiyamaella* forms a well-supported monophyletic group, distinct from *Spencermartinsiella* and *Diddensiella*. However, Sena et al. (2017) indicated that *Sugiyamaella* is polyphyletic, where the species are intertwined with representatives of the genera *Trichomonascus* and *Spencermartinsiella*. The results of our phylogenetic analyses of combined gene sequences (ITS and nr LSU) with all currently-known species indicated that the genus is not monophyletic and grouped into a paraphyletic grade with three well-supported clades (Fig. 1): (i) *S. smithiae* (the type species), *S. lignohabitans* and *S. valdiviana* and their related species, (ii) *S. ayubii*, *S. trypani*, *S. valenteae* and *S. chuxiong* (described in this paper) and (iii) *S. americana*, *S. bullrunensis*, *S. carassensis* and *S. ligni*. These results suggest that the genus *Sugiyamaella* should be limited to species of the clade comprising the type species *S. smithiae*. The remaining two clades, which have previously been considered members of *Sugiyamaella*, could become two novel genera, although their phylogenetic relationships with other genera were not fully examined by this study (Fig. 1). As such, a careful phylogenetic analysis of *Sugiyamaella* species is required to clarify the possible heterogeneity of the genus.

Many new yeast species have been identified in the last ten years in China (Wang et al. 2010; Liu et al. 2016; Huang et al. 2018; Zhai et al. 2019). However, there is still a large number of undescribed yeast taxa in this country. This study indicates that there are at least 12 species of *Sugiyamaella* in China, including four species known previously to occur in China (*S. lignohabitans*, *S. qingdaonensis*, *S. smithiae* and *S. xiaguanensis*), new records of six species not known to occur in China (*S. americana*, *S. ayubii*, *S. novakii*, *S. paludigena*, *S. valenteae* and *S. valdiviana*) and two novel species (*S. chuxiong* and *S. yunanensis*). In China, there are still some species that need to be discovered, such as that listed under GenBank accession JN581116. To date, including the two novel species described in this study, there are thirty-one species of *Sugiyamaella* worldwide. Although the taxonomy of *Sugiyamaella* has received much attention in the past, many regions in China are under-sampled and more under-described indigenous *Sugiyamaella* species will undoubtedly be discovered in the future.

*Sugiyamaella* species have a worldwide distribution and are isolated from a wide range of substrates. Insect is their main habitat, but new species were also isolated from frass, rotting wood, decayed log, forest soil, mushrooms and peat (Kurtzman 2007; Wang et al. 2010; Kurtzman 2011; Morais et al. 2013; Handel et al. 2016; Sena et al. 2017; Huang et al. 2018). These studies expanded our knowledge on the substrates where *Sugiyamaella* species can occur, but on the other hand, demonstrated the complicated ecological function of this genus. In this study, seven known species and two new species were identified from rotting wood in China. Further research will focus on the *Sugiyamaella* diversity from a wide range of substrates.

## Supplementary Material

XML Treatment for Sugiyamaella yunanensis

XML Treatment for Sugiyamaella chuxiongensis
